# The effect of red light and far-red light conditions on secondary metabolism in Agarwood

**DOI:** 10.1186/s12870-015-0537-y

**Published:** 2015-06-12

**Authors:** Tony Chien-Yen Kuo, Chuan-Hung Chen, Shu-Hwa Chen, I-Hsuan Lu, Mei-Ju Chu, Li-Chun Huang, Chung-Yen Lin, Chien-Yu Chen, Hsiao-Feng Lo, Shih-Tong Jeng, Long-Fang O. Chen

**Affiliations:** Institute of Plant and Microbial Biology, Academia Sinica, 128 Sec. 2, Academia Rd, 11529 Nankang, Taipei Taiwan; Department of Bio-industrial Mechatronics Engineering, National Taiwan University, Taipei, 106 Taiwan; Institute of Plant Biology, College of Life Science, National Taiwan University, Taipei, 106 Taiwan; Institute of Information Science, Academia Sinica, Taipei, 115 Taiwan; Division of Biostatistics and Bioinformatics, Institute of Population Health Sciences, National Health Research Institutes, Zhunan, 350 Taiwan; Institute of Fisheries Science, College of Life Science, National Taiwan University, Taipei, 106 Taiwan; Center for Systems Biology, National Taiwan University, Taipei, 106 Taiwan; Department of Horticulture and Landscape Architecture, National Taiwan University, Taipei, 106 Taiwan

**Keywords:** Agarwood, Aquilaria agallocha, Genome, Secondary metabolism, Red light, Cucurbitacin

## Abstract

**Background:**

Agarwood, a heartwood derived from *Aquilaria* trees, is a valuable commodity that has seen prevalent use among many cultures. In particular, it is widely used in herbal medicine and many compounds in agarwood are known to exhibit medicinal properties. Although there exists much research into medicinal herbs and extraction of high value compounds, few have focused on increasing the quantity of target compounds through stimulation of its related pathways in this species.

**Results:**

In this study, we observed that cucurbitacin yield can be increased through the use of different light conditions to stimulate related pathways and conducted three types of high-throughput sequencing experiments in order to study the effect of light conditions on secondary metabolism in agarwood. We constructed genome-wide profiles of RNA expression, small RNA, and DNA methylation under red light and far-red light conditions. With these profiles, we identified a set of small RNA which potentially regulates gene expression via the RNA-directed DNA methylation pathway.

**Conclusions:**

We demonstrate that light conditions can be used to stimulate pathways related to secondary metabolism, increasing the yield of cucurbitacins. The genome-wide expression and methylation profiles from our study provide insight into the effect of light on gene expression for secondary metabolism in agarwood and provide compelling new candidates towards the study of functional secondary metabolic components.

**Electronic supplementary material:**

The online version of this article (doi:10.1186/s12870-015-0537-y) contains supplementary material, which is available to authorized users.

## Background

Agarwood is resinous heartwood derived from *Aquilaria* and *Gyrinops* trees. Due to the high economic value of these trees and the extensive deforestation, agarwood producing tree species have become endangered. The use of agarwood is prevalent in many cultures for religious ceremonies, perfumes, and especially in Chinese herbal medicine, where plant materials are commonly utilized [1, 2]. Agarwood is one of the most used plant materials in Chinese medicine, second only to ginseng. The value of agarwood lies not only in its aromatic compounds [3], but also in its non-volatile compounds, which potentially have beneficial properties with regards to human medicine [4, 5].

In our previous study, we presented a draft genome and a putative pathway for cucurbitacins E and I, compounds with known medicinal value, in *Aquilaria agallocha* [6], one of the largest producers of agarwood. Briefly, gene expression changes for in vitro samples treated with methyl jasmonate (MJ) were shown to be consistent with known responses of *A. agallocha* to biotic stress and a set of homologous genes related to cucurbitacin biosynthesis in *Arabidopsis thaliana* was identified. However, MJ treatment is perhaps not the most efficient protocol. Although there exists much research into Chinese medicinal herbs and extraction of high value compounds, few have focused on increasing the quantity of target compounds through stimulation of its related pathways in this species.

In this study, we demonstrate that the quantity of cucurbitacins can be controlled by utilizing different types of light. Red light (R) and far-red light (FR) are components of the solar spectrum that strongly affect plant tissues. Many studies have reported an interaction between plant defenses and R/FR responses [7, 8]. Under low R/FR conditions, there is a dramatic decrease not only in the number of root nodules but also in the expression of jasmonic acid (JA) response genes. In a study on phytochrome B (*phyB*) mutants, JA-related gene expression levels have also been observed to be down-regulated [9] and are known to participate in secondary metabolic pathways [10].

In order to better understand the effect of light conditions on cucurbitacin secondary metabolic pathways in *A. agallocha*, we performed high-throughput sequencing experiments under two different light conditions: red light, a factor activating *phyB*, and far-red light, a factor inhibiting *phyB* [11]. Three types of sequencing experiments were performed: RNA sequencing (RNA-seq) to study gene expression, whole-genome bisulfite sequencing to study DNA methylation, and small RNA (sRNA) sequencing to determine sRNAs that play a role in methylation. As epigenetic modifications may also play a role in the regulation of gene expression, studies on DNA methylation are becoming increasing important.

To higher organisms, DNA methylation plays an important and widespread role in epigenetic modification, mediated by DNA methyltransferases (DMTs). DNA methylation in the genome is known to provide protection from transposons and/or RNA viruses, where they play a role in regulating splicing. DNA methylation is also associated with major developmental reprogramming [12]. Small RNAs are also an essential factor in plants where they play a role in regulating the activation of functional genes and transposons [3].

The results of our analysis show that R/FR conditions have a large effect on gene expression levels in agarwood. RNA-seq data revealed an array of gene clusters with distinctive expression patterns, where individual gene clusters responded primarily to red light or far-red light. Differentially methylated regions (DMRs) discovered from whole-genome bisulfite sequencing data showed that there is also a large difference in methylation levels between R/FR conditions. We observed that sRNAs may potentially play a role in influencing the methylation levels of genes important to secondary metabolism and subsequently play a role in gene expression regulation.

These genome wide profiles provide insight into the regulatory interaction between red light and far-red light conditions in *A. agallocha* as well as identify compelling new candidates for secondary metabolic functional components. The data used in this study is freely available at our provided webserver (http://molas.iis.sinica.edu.tw/agarwood) and at NCBI (Bioproject ID: PRJNA240626).

## Results and discussion

### Red light conditions increase cucurbitacin E and I content

**Fig. 1:**
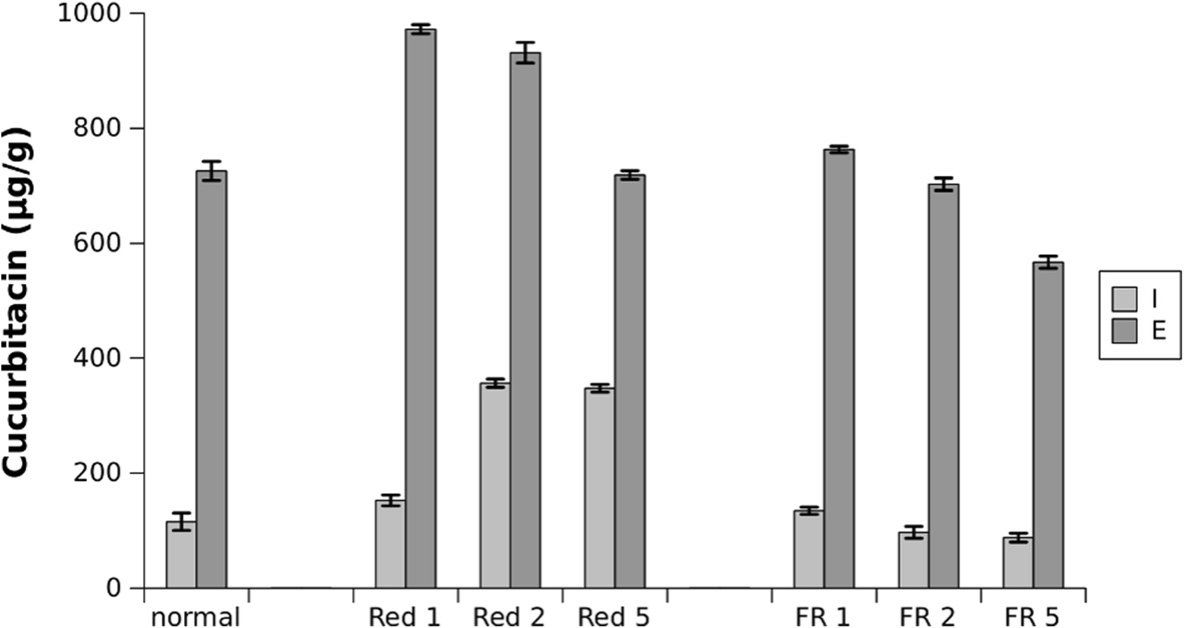
Endogenous cucurbitacin content of in vitro agarwood. Content was measured after red and far-red light treatment over the course of 5 days. Data is represented as mean ± standard deviation (n = 5). At peak levels under red light conditions, cucurbitacin content was significantly increased compared to normal light conditions (paired *t*-test p-values 1.09E-5 and 4.57E-6 for cucurbitacin I and E respectively). At the lowest levels under far-red light conditions, cucurbitacin content was significantly decreased compared to normal light conditions (paired *t*-test p-values 3.44E-2 and 1.32E-4 for cucurbitacin I and E respectively)

In our previous study, we showed that agarwood contained high cucurbitacin content and that MJ treatment increased content levels [6]. Here, we instead used red light conditions to stimulate cucurbitacin biosynthesis (Fig. 1). From LC-ESI-MS quantification, it was seen that cucurbitacin content increased as red light exposure increased, up to 356 μg/g of cucurbitacin I at day 2. Cucurbitacin I content decreased as far-red light exposure increased, down to 96 μg/g at day 2. Similarly for cucurbitacin E, content levels increased up to 972 μg/g under red light conditions at day 1 and decreased down to 567 μg/g under far-red light conditions at day 5. Under red light conditions, at peak levels, cucurbitacin content was significantly increased compared to normal light conditions with p-values of 1.09E-5 and 4.57E-6 for cucurbitacin I and E respectively in a two-sample *t*-test. Similarly for far-red light conditions, at the lowest levels, cucurbitacin content was significantly decreased compared to normal light conditions with p-values of 3.44E-2 and 1.32E-4 for cucurbitacin I and E respectively.

Different types of light affect various biological pathways in plants. There are five classes of phytochromes which typically absorb red light and far-red light [13]. Previous studies on *phyA* and *phyB* photosensory functions show that red light activated *phyB* interacts with transcription factors to induce a phytochrome-dependent signaling cascade [7, 8] and that vascular plant one-zinc-finger (VOZ) transcription factors interact with *phyB* [14]. VOZs are active transcription factors that promote SA and JA-mediated defense responses under biotic stress [14, 15]. Far-red light is known to inhibit *phyB* and plays an antagonistic role in most pathways [11, 14].

Previous studies have demonstrated that target compounds can be increased through stimulating biosynthetic pathways [6, 16] and that light can be used as stimuli for increasing compound yield [17]. With the increasing commonality of plant factories, the use of light as stimuli instead of chemical treatment may be preferable due to a simpler protocol.

### Red light and far-red light gene expression patterns in agarwood

In order to study the effects of different light on gene expression in agarwood, we performed high-throughput RNA sequencing under red light and far-red light conditions. The time-course RNA-seq data (Table 1) was obtained from samples under red light and far-red light conditions at 1, 2, and 5 days, as well as normal conditions (white light control). Two biological replicates were sequenced.

**Table 1:** RNA-seq libraries under different light conditions

		Replicate 1	Replicate 2
Sample	Read Length	No. Read Pairs	No. Read Pairs
Normal	91 bp	27,666,839	25,224,541
FR day 1	91 bp	27,019,309	25,348,603
FR day 2	91 bp	24,633,314	28,295,864
FR day 5	91 bp	21,851,783	27,796,463
R day 1	91 bp	26,495,438	29,309,684
R day 2	91 bp	24,259,434	39,811,543
R day 5	91 bp	23,467,848	27,372,435

**Fig. 2:**
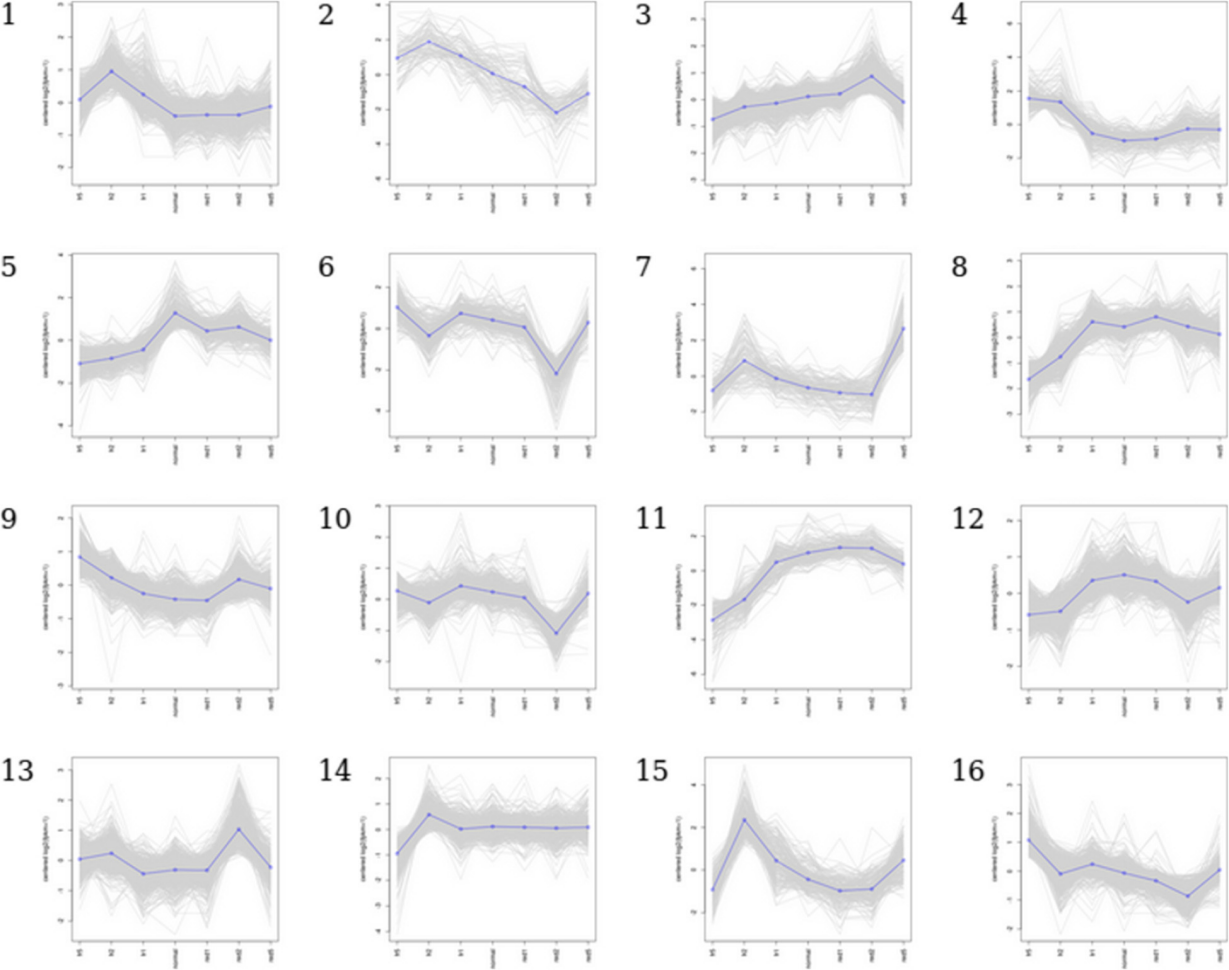
Cluster analysis of gene expression patterns in agarwood. Sixteen clusters were identified by k-means clustering. The samples are represented on the x-axis, from left to right: FR day 5, FR day 2, FR day 1, normal, R day 1, R day 2, R day 5. The centered log2 fold-change is represented on the y-axis

We utilized the RNA-seq data and the previously constructed *A. agallocha* genome [6] for gene expression quantification, resulting in an average correlation coefficient of 0.9404 for gene expression levels between biological replicates. Genes were clustered into 16 clusters based on their expression patterns, requiring a two-fold change in expression and a p-value cut-off of 0.001 for differential expression (Fig. 2). In total, 8882 genes were determined to be differentially expressed and clustered into distinct expression patterns (Additional file 1: Table S1). Gene ontology (GO) classification was performed to identify each cluster’s most significant biological process (Table 2).

**Table 2:** Gene ontology analysis on 16 clusters of gene expression patterns

Cluster	No. Terms	Most Significant Term	p-value
1	82	lipid glycosylation	1.04E-03
2	14	negative regulation of nucleotide metabolic process	4.73E-03
3	143	cell redox homeostasis	8.23E-06
4	28	trehalose biosynthetic process	2.25E-03
5	85	oxidation-reduction process	1.42E-07
6	62	protein import	1.61E-03
7	26	oxidation-reduction process	1.23E-05
8	86	photosynthesis	9.88E-05
9	112	translational termination	1.90E-04
10	146	vesicle-mediated transport	4.50E-04
11	38	photosynthesis	6.21E-05
12	130	biosynthetic process	7.39E-06
13	115	response to water stimulus	4.93E-03
14	100	proteolysis involved in cellular protein catabolic process	2.60E-04
15	39	oxidation-reduction process	2.06E-03
16	140	detection of visible light	4.60E-04

Clusters 3 and 11 were observed to exhibit a pattern of up-regulation under red light conditions and repression under far-red light conditions, consistent with the observed changes in cucurbitacin content levels. The GO classifications show that 253 out of 495 genes, in clusters 3 and 11 combined, are classified as belonging to metabolic processes (Additional file 2: Figure S1). Furthermore, these clusters contain 3 genes classified as belonging to terpene biosynthesis, the main class of compounds related to the medicinal properties of agarwood [18–20]. Terpenoid content is induced under biotic stress as an immune response to resist various pathogens [6, 21] and its derivatives have been shown to exhibit anti-microorganism, anti-tumour, and other pharmacological effects that are beneficial towards human medicine [4, 5]. In addition to terpene biosynthesis, clusters 3 and 11 contained 26 genes related to defense response. Previous studies have shown that far-red light down-regulates the expression of defense response genes by reducing a plant’s sensitivity to jasmonate (or methyl jasmonate) in *Arabidopsis* [7, 8]. From the RNA-seq data, it was seen that some defense response genes were up-regulated under red light conditions and down-regulated under far-red light conditions. These results are consistent with our expectations and suggest that controlled light conditions can be used in place of plant hormones to induce defense response genes in agarwood.

### Red light and far-red light DNA methylation patterns in agarwood

**Fig. 3:**
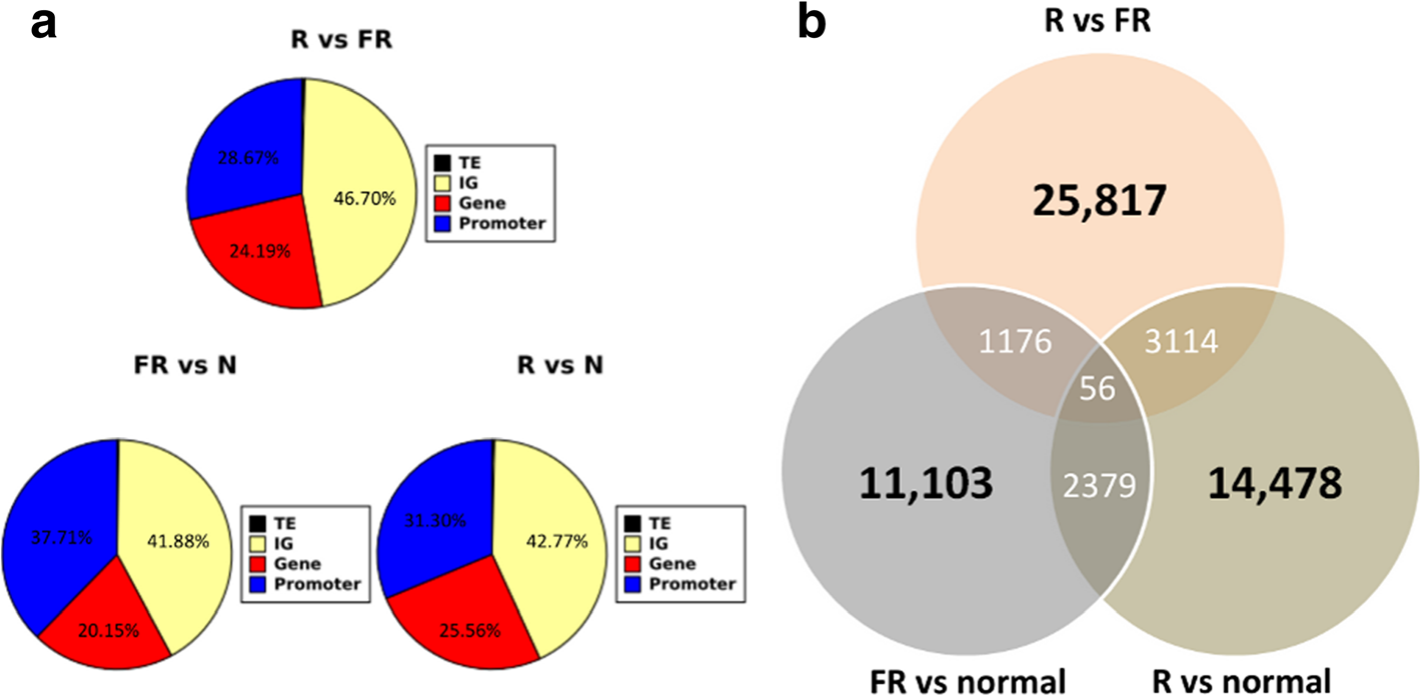
Characterization of differentially methylated regions for light conditions red light, far-red light, and normal. **a** Composition of DMRs in the *A. agallocha* genome. TE represents transposable elements, IG represents intergenic regions, Gene represents the gene body, and Promoter represents gene promoter regions. **b** Number of DMRs that are overlapping or unique to red light and far-red light conditions

In order to study the effect of different light on methylation patterns in agarwood, we performed whole-genome bisulfite sequencing with two biological replicates for red light day 2, far-red light day 2, and normal samples (Additional file 2: Table S2). The methylation levels for each sample were used to discover differentially methylated regions (DMR) between different light conditions. A characterization of DMRs (Fig. 3a) shows that DMR proportions in transposons and intergenic regions were not significantly changed by R or FR conditions. In genic regions, it was seen that there was a slight increase (~6.4 %) in DMR proportions at promoter regions under FR conditions. The number of DMRs for each light condition (Fig. 3b) indicates that there is a large change in methylation levels between red light and far-red light conditions.

**Fig. 4:**
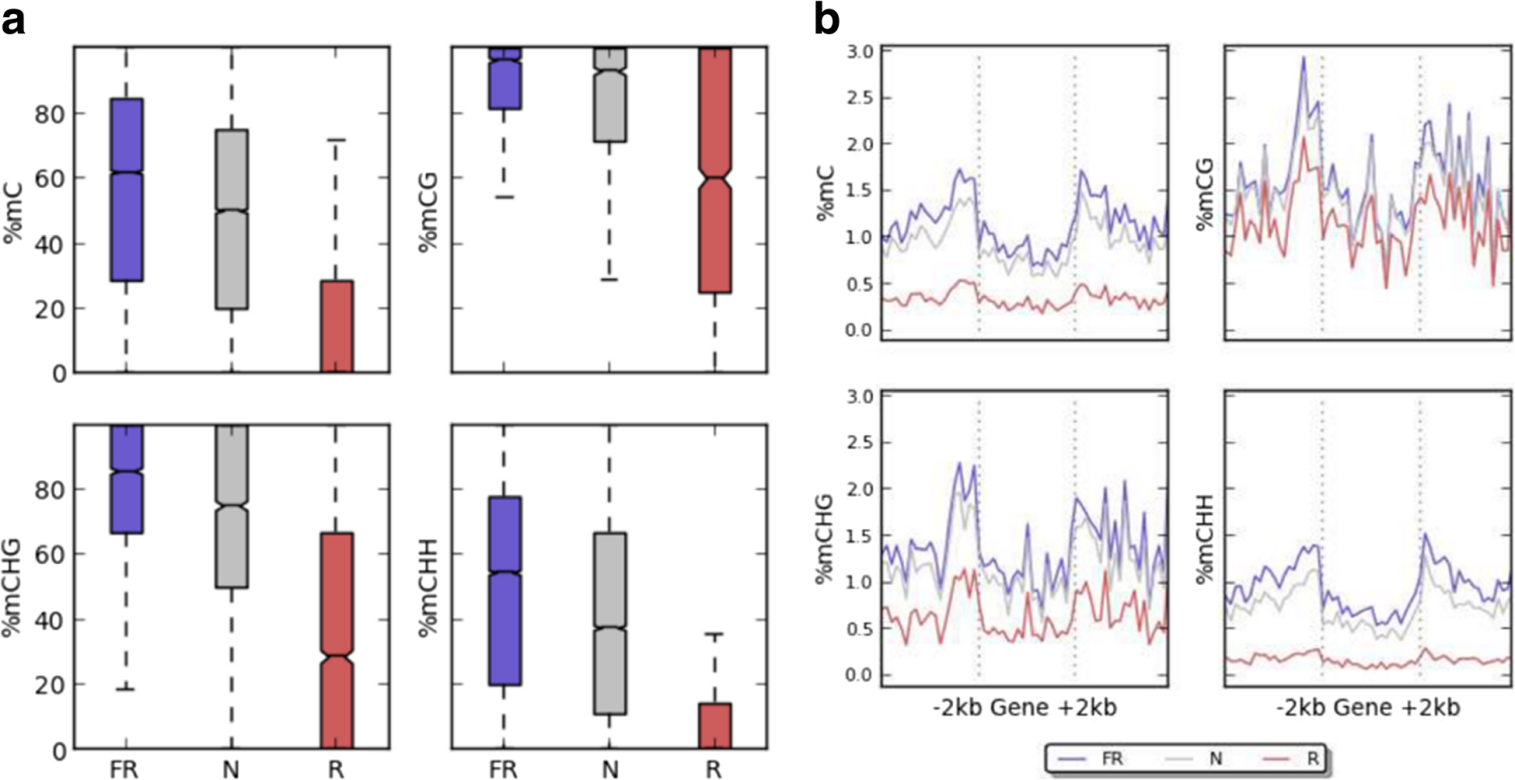
Methylation levels for hypo-DMRs under red light conditions. **a** Box plots displaying the distribution of average CG, CHG, and CHH methylation levels for hypo-DMRs under red light conditions. **b** Average methylation levels in gene bodies and flanking 2 kb regions. Each gene was aligned from start to end and divided into 20 equal bins. Upstream and downstream flanking regions were also each divided into 20 equal bins. Weighted methylation levels were calculated for each of the 60 bins across all corresponding regions

We focused on hypo-DMRs under red light conditions, using the consensus hypo-DMRs between R/normal and R/FR data, resulting in 621 regions for analysis. The average methylation levels in red light hypo-DMRs (Fig. 4a) show that CHH methylation (where H represents A, T, or C) exhibit the most significant differences under red light conditions. This remains the trend for average weighted methylation levels [22] in genic regions (Fig. 4b), where the most significant differences in methylation levels were observed in promoter regions for CHH methylation. CHG methylation levels were also observed to be affected by red light while CG methylation levels were relatively unchanged. These results suggest that red light may regulate gene expression in agarwood by changing CHH and CHG methylation, primarily in promoter regions.

In higher plants, Domains Rearranged Methylase 2 (DRM2) catalyzes *de novo* DNA methylation in all cytosine contexts including CG, CHG, and CHH [23], via the RNA-directed DNA methylation pathway (RdDM) [24–26]. Cytosine methylation and demethylation are both closely linked with gene regulation where high methylation patterns typically accompany low gene expression [27, 28]. In RdDM, Argonaute 4 (AGO4) has been recognized to interact with sRNAs and participate in DNA methylation [28–30].

### sRNAome of red light and far-red light conditions in agarwood

In order to identify sRNAs that play a role in changes to methylation under different light conditions, we performed sRNA sequencing with two biological replicates for red light day 2, far-red light day 2, and normal samples (Table S2). Overall, approximately 6 million distinct sRNAs were able to be mapped perfectly and uniquely to the genome. A characterization of mapped sRNAs (Additional file 2: Figure S2) revealed that the majority (56.28 %) of sRNAs were mapped to genic regions, within which, a large majority (61.11 %) were mapped to promoter regions. As well, we characterized the mapped sRNAs in terms of their length (Table 3) and observed that 71.93 % of the sRNAs were 24-nt long overall, 73.37 % in promoter regions. These results support the idea that under different light conditions, sRNA may play a role in DNA methylation via AGO4 and the RdDM pathway in agarwood.

**Table 3:** Characterization of sRNAs by sequence length

Length	Mapped	Intergenic	Genic	Promoter	G. Body	Intron	Exon
20 bp	114,233	41,928	72,305	35,197	37,108	11,712	25,396
21 bp	553,289	205,532	347,757	172,777	174,980	52,227	122,753
22 bp	253,020	88,252	164,768	73,132	91,636	27,515	64,121
23 bp	537,380	232,440	304,940	195,293	109,647	49,544	60,103
24 bp	4,387,009	2,001,459	2,385,550	1,539,302	846,248	387,823	458,425
25 bp	220,819	90,824	129,995	77,841	52,154	20,234	31,920
26 bp	18,290	3,972	14,318	3,062	11,256	1,868	9,388
27 bp	8,895	1,196	7,699	743	6,956	966	5,990
28 bp	4,356	553	3,803	291	3,512	531	2,981
29 bp	1,465	193	1,272	80	1,192	163	1,029
30 bp	413	61	352	31	321	46	275

Small RNAs are classified into two major categories: microRNA (miRNA) and short interfering RNA (siRNA) [31]. Small RNAs, which are cut from double-stranded RNA (dsRNA) by Dicer-like enzymes, participate in gene silencing as miRNA [32–34]. The focus of this study, siRNAs, are processed from the overlapping regions of natural sense-antisense transcript pairs or the near-perfect double-stranded RNAs (dsRNAs) synthesized by RNA-dependent RNA polymerases (RDRs) [35–37]. Based on their origins, plant siRNAs include four major classes: heterochromatic siRNAs (hc-siRNAs), trans-acting siRNAs (ta-siRNAs), natural antisense transcript-derived siRNAs (nat-siRNAs), and long siRNAs (lsiRNAs) [38]. siRNAs bind to specific Argonaute proteins to form a RNA-induced silencing complex (RISC) guiding RISCs to DNA or RNA targets based on sequence complementarity and trigger gene silencing transcriptionally or post-transcriptionally [31]. Different AGOs have different preferences. AGO1 has a strong bias towards 5’ terminal uridine, AGO2 prefers 5’ terminal adenosine, and AGO4 prefers 5’ terminal adenosine, guanine, or uridine [29]. Different length small RNAs play different roles and are cut by different Dicer-like enzymes (DCL) [34, 36, 39]. Among them, the 24-nt long miRNAs (lmiRNAs) and 24-nt siRNAs are processed by DCL3 [40]. These 24-nt small RNAs interact with AGO4 and acts as a guide to catalyze DNA methylation via RdDM [40, 41].

### Regulation of secondary metabolic gene expression by RdDM pathway

Although DNA methylation in promoter regions and intergenic transposable elements generally inhibit gene expression [42], the role of DNA methylation in *A. agallocha* is still unclear. To further our understanding of DNA methylation in *A. agallocha*, we identified sRNAs that inhibit gene expression through the RdDM pathway selected from the set of metabolic processes genes containing hypo-methylated regions (Additional file 2: Figure S3).

**Fig. 5:**
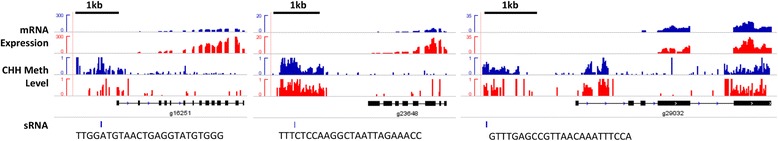
Light conditions regulate gene expression by the RdDM pathway. The RNA expression, DNA methylation, and sRNA expression is shown for three candidate genes: g16251 (sterol methytransferase), g23648 (hydroxysteroid dehydrogenase), and g29032 (cytochrome P450). Signals in red represent red light conditions while signals in blue represent far-red light conditions

As mentioned previously, different AGOs have different preferences. Here, we focused on sRNA sequences that suited AGO4 preferences and mapped to hypo-DMRs. We identified 61 genes in agarwood related to secondary metabolism that fit our criteria. Three candidate genes were selected for further analysis (Fig. 5), a sterol methytransferase (g16251), a hydroxysteroid dehydrogenase (g23648), and a cytochrome P450 (g29032). The selected genes show that sRNAs were mapped to red light hypo-DMRs with a corresponding increase in mRNA expression under red light conditions. The expression levels were also verified using qRT-PCR (Additional file 2: Figure S4).

In the three candidate genes, we detected three specific sRNAs that mapped perfectly to promoter regions under far-red light conditions. It was seen that these sRNAs had a positive relationship with DNA methylation levels and a negative relationship with gene expression levels. In contrast, for both the sRNA sequencing and qRT-PCR validation, these sRNAs were not able to be detected under red light conditions. This suggests that the effects of red light and far-red light on secondary metabolism gene expression in agarwood are antagonistic to each other and that these sRNAs potentially play a role in gene expression regulation through the RdDM pathway in cucurbitacin biosynthesis.

Sterols (steroid alcohols) belong to steroids and are ubiquitous in eukaryotic organisms, playing pivotal roles in membrane structure and as precursors of vitamins and steroid hormones [43]. Sterol methyltransferases are known to catalyze a single methyl addition, an important step in phytosterol synthesis [43], and important to biosynthesis of secondary metabolites such as cucurbitacin. Hydroxysteroid dehydrogenases belong to alcohol oxidoreductases, which catalyzes the dehydrogenation of hydroxysteroid in steroidgenesis by cofactor NADP(H) or NAD and may affect the activity of compounds [44]. Cytochrome P450s (CYP450s) are also ubiquitous in many organisms. In plants, one or more CYP450s participate in compound modification and affect compound activity in secondary metabolism [45]. As well, some CYP450s play an important role in steroidgenesis [46, 47].

Although these three candidate genes belong to rather large gene families, the gene expression, sRNA, and methylation patterns under red light and far-red light conditions indicate that these genes are potentially important for cucurbitacin metabolism in agarwood.

## Conclusion

In this study, we performed three types of sequencing experiments in order to study the effect of light conditions on cucurbitacin biosynthesis and secondary metabolism in agarwood. This resulted in a number of new insights regarding the global regulation of genes by red light and far-red light. From the RNA sequencing results, gene expression patterns were clustered into distinct clusters, many of which can be characterized as responding primarily to light conditions. In particular, two gene expression clusters clearly exhibited gene expression patterns in response to red light and far-red light. Significantly, the two clusters included genes related to terpene biosynthesis and defense response. In addition to gene expression, small RNA and DNA methylation were observed to be factors affected by different light conditions which in turn affect cucurbitacin metabolism in agarwood. We identified a set of small RNA which potentially regulates gene expression through the RdDM pathway.

The results from this study provide genome-wide profiles of RNA expression, small RNA, and DNA methylation with regards to light conditions. These profiles provide insight into the effect of light on gene expression for cucurbitacin biosynthesis in agarwood as well as provide compelling new candidates for functional secondary metabolic components, highlighting new questions to be addressed in future studies.

We also demonstrate that light conditions can be used in lieu of methyl jasmonate treatment to stimulate pathways related to secondary metabolism, increasing the yield of cucurbitacins. This has important implications for the increasing use of plant factories for the synthesis of high value compounds.

## Methods

### Plant materials for DNA and RNA extraction

A plant regeneration system from shoot tips into in vitro plants was created using a tissue culture process similar to the processes described by He et al. [48]. LED light sources (Daina Electronics) were used to provide different light conditions (Table S3). Normal (white light ~55 μmol m^−2^ s^−1^) in vitro plant materials were grown under long-day conditions (16 h of light, 8 h of darkness) at 25 °C. Red light samples (~15 μmol m^−2^ s^−1^, 680 nm) and far-red light samples (~15 μmol m^−2^ s^−1^, 730 nm) were continuously exposed to their respective light conditions at 25 °C and the materials used for sequencing were collected after 1, 2, and 5 days.

DNA was extracted from 1 g of in vitro materials using the Plant Genomic DNA MiniKit (Maestrogen) following the manufacturer’s instructions. RNA was extracted from 1 g of in vitro materials using RNeasy Plant MiniKit following the protocol prescribed by the manufacturer. Normal light samples were collected from material grown under long-day conditions in white light. The DNA and RNA samples were sent to BGI for poly(A) RNA sequencing, whole-genome bisulfite sequencing, and small RNA sequencing.

### LC-ESI-MS

In vitro materials were ground with liquid nitrogen and mixed with 1 mL of methanol. Supernatant was collected by centrifugation (12000 rpm, 1 min). The LC-ESI-MS system consisted of an ultra-performance liquid chromatography system (Ultimate 3000 RSLC, Dionex) and an electrospray ionization source of quadrupole time-of-flight mass spectrometer (maXis HUR-QToF system, Bruker Daltonics). The autosampler was set at 4 °C. Separation was performed with reversed-phase liquid chromatography on a BEH C8 column (2.1 × 100 mm, Walters). The elution started from 99 % mobile phase A (0.1 % formic acid in ultrapure water) and 1 % mobile phase B (0.1 % formic acid in ACN), held at 1 % B for 1.5 min, raised to 60 % B in 6 min, further raised to 90 % in 0.5 min, and then lowered to 1 % B in 0.5 min. The column was equilibrated by pumping 1 % B for 4 min. The flow rate was set to 0.4 mL/min with an injection volume of 5 μL. LC-ESI-MS chromatogram were acquired under the following conditions: capillary voltage of 4500 V in positive ion mode, dry temperature of 190 °C, dry gas flow maintained at 8 L/min, nebulizer gas at 1.4 bar, and acquisition range of m/z 100–1000. Five samples for each condition were independently measured for cucurbitacin content levels.

### RNA sequencing analysis

The RNA-seq data for all samples (Table 1) were trimmed for low quality bases at the 3’ terminal and then individually aligned to the set of annotated *A. agallocha* transcripts using BWA [49]. For each dataset, expression quantification was performed using eXpress [50]. R/FR pair-wise differential gene expression analysis was performed using edgeR [51] incorporating all replicates. Genes which exhibit at least a two-fold change in expression with a p-value threshold of 0.001 between any red light and far-red light sample were retained for clustering analysis. Clustering analysis was performed on the expression profiles of differentially expressed genes using k-means clustering. Gene ontology classifications for each cluster was performed using BinGO [52].

### Whole-genome bisulfite sequencing analysis

The whole-genome bisulfite sequencing data for red light day 2, far-red light day 2, and normal were trimmed for low quality bases at the 3’ terminal. MOABS [53] was utilized to perform alignment to the *A. agallocha* genome, methylated cytosine calling, discovery of differentially methylated cytosines (DMCs), and discovery of differentially methylated regions (DMRs). Differentially methylated cytosines were discovered using a Fisher Exact Test, with a p-value threshold of 0.05, a minimum depth of 3, and a minimum of 33 % nominal difference in methylation ratios between conditions. Differentially methylated regions were discovered using a Fisher Exact Test, with a p-value threshold of 0.05, a minimum of 3 DMCs in a region, and a maximum distance of 300 bp between DMCs.

### sRNA sequencing analysis

The sRNA sequencing reads for red light day 2, far-red light day 2, and normal were aligned to the *A. agallocha* genome using BWA [49]. Only sequences with perfect mappings (no mismatches, no gaps) and uniquely mapped (to one genome location only) were retained for analysis.

### qRT-PCR analysis

Validation of RNA expression on three candidate genes was performed using qRT–PCR analysis. The RNA samples for each light condition were extracted from 1 g of in vitro *A. agallocha* shoots using RNeasy Plant MiniKit following the protocol prescribed by the manufacturer. Primers pairs were designed for each transcript (Table S4) with the ABI Prism 7500 sequence detection system (Applied Biosystems). Each primer pair was used to amplify the respective cDNA fragments using a cycling profile consisting of 58 °C for 2 min, 95 °C for 10 min, and 40 cycles of 95 °C for 15 s and 60 °C for 1 min. The relative gene expression was determined by the comparative CT method, 2^−ΔCT^ (ΔC_T_ = C_T_, gene of interest – C_T_, control gene), using *AcHistone* as the internal control [54]. Four independent biological repeats were performed for each assay where the final expression value is the mean expression of the repeats.

Validation of sRNA used the same plant materials as described above. An endogenous sRNA (CGGTGGAAGAAATAATAGGGCCTG) was chosen as internal control due to its expression levels being stable under different light conditions (mean TPM of 237.00 ± 39.44) as well as uniquely mapping to an intergenic region and thus will not affect genes. For detecting sRNAs of g16251, g23648, and g29032, miScript Primer Assays (Qiagen) #MSC0074731, #MSC0074729, and #MSC0074727, respectively, as well as the miScript Universal primer were used. Five independent biological repeats were performed for each assay where the final expression value is the mean expression of the repeats.

### Availability of supporting data

The datasets supporting the results of this article are available in the NCBI repository, BioProject ID: PRJNA240626, http://www.ncbi.nlm.nih.gov/bioproject/?term=PRJNA240626. Gene annotations, KEGG, and GO classifications for *Aquilaria agallocha* are available at our webserver, http://molas.iis.sinica.edu.tw/agarwood.

## Additional files

Additional file 1: Table S1. The set of genes in each gene expression cluster. Additional file 2: Table S2. (a) Whole-genome bisulfite sequencing DNA libraries and (b) sRNA sequencing libraries. **Table S3.** Spectral data of lamps used for different light conditions in this study. **Table S4.** Gene specific primers for real-time PCR analysis of gene expression. **Figure S1.** Gene Ontology classifications of the set of transcripts in cluster 3 and cluster 11. Relative gene proportions were calculated separately for Biological Process and Molecular Function. **Figure S2.** The composition of sRNAs that mapped to the *A. agallocha* genome. Only sRNAs which mapped perfectly and uniquely to one genome location were retained for analysis. **Figure S3.** Gene Ontology classifications of hyper and hypo differentially methylated regions. Relative gene proportions were calculated separately for Biological Process and Molecular Function. The set of metabolic process genes containing hypo-methylated regions were curated for secondary metabolic function and sRNA which mapped to hypo-DMR regions. **Figure S4.** qRT-PCR validation of mRNA expression and sRNA expression. Expression quantification from sequencing data as FPKM and TPM of the mRNA and sRNA expression are also shown, respectively.

## Abbreviations

AGO4: Argonaute 4
CYP450s: Cytochrome P450s
DCL: Dicer-like enzyme
DMRs: Differentially methylated regions
DMTs: DNA methyltransferases
DRM2: Domains rearranged Methylase 2
dsRNA: Double-stranded RNA
DMCs: Differentially methylated cytosines
FR: Far-red light
hc-siRNAs: Heterochromatic siRNAs
GO: Gene ontology
lsiRNAs: Long siRNAs
lmiRNAs: Long miRNAs
JA: Jasmonic acid
*phyB*: Phytochrome B
nat-siRNAs: Natural antisense transcript-derived siRNAs
MJ: Methyl jasmonate
miRNA: MicroRNA
R: Red light
RNA-seq: RNA sequencing
RdDM: RNA-directed DNA methylation pathway
RDRs: RNA-dependent RNA polymerases
RISC: RNA-induced silencing complex
sRNA: Small RNA
siRNA: Short interfering RNA
ta-siRNAs: Trans-acting siRNAs

## References

[1] Chen KJ, Yu B (1999). Certain progress of clinical research on Chinese integrative medicine. Chinese Med J-Peking.

[2] Shang AJ, Huwiler K, Nartey L, Juni P, Egger M (2007). Placebo-controlled trials of Chinese herbal medicine and conventional medicinecomparative study. Int J Epidemiol.

[3] Kumeta Y, Ito M (2010). Characterization of delta-guaiene synthases from cultured cells of Aquilaria, responsible for the formation of the sesquiterpenes in agarwood. Plant Physiol.

[4] Chen H, Yang Y, Xue J, Wei J, Zhang Z (2011). Comparison of compositions and antimicrobial activities of essential oils from chemically stimulated agarwood, wild agarwood and healthy Aquilaria sinensis (Lour.) gilg trees. Molecules.

[5] Momma K, Masuzawa Y, Nakai N, Chujo M, Murakami A, Kioka N (2008). Direct interaction of Cucurbitacin E isolated from Alsomitra macrocarpa to actin filament. Cytotechnology.

[6] Chen CH, Kuo TC, Yang MH, Chien TY, Chu MJ, Huang LC (2014). Identification of cucurbitacins and assembly of a draft genome for Aquilaria agallocha. BMC Genomics.

[7] Izaguirre MM, Mazza CA, Biondini M, Baldwin IT, Ballare CL (2006). Remote sensing of future competitors: impacts on plant defenses. Proc Natl Acad Sci U S A.

[8] Moreno JE, Tao Y, Chory J, Ballare CL (2009). Ecological modulation of plant defense via phytochrome control of jasmonate sensitivity. Proc Natl Acad Sci U S A.

[9] Suzuki A, Suriyagoda L, Shigeyama T, Tominaga A, Sasaki M, Hiratsuka Y (2011). Lotus japonicus nodulation is photomorphogenetically controlled by sensing the red/far red (R/FR) ratio through jasmonic acid (JA) signaling. Proc Natl Acad Sci U S A.

[10] Bennett RN, Wallsgrove RM (1994). Secondary Metabolites in Plant Defense-Mechanisms. New Phytol.

[11] Quail PH, Boylan MT, Parks BM, Short TW, Xu Y, Wagner D (1995). Phytochromes: photosensory perception and signal transduction. Science.

[12] Finnegan EJ, Peacock WJ, Dennis ES (1996). Reduced DNA methylation in Arabidopsis thaliana results in abnormal plant development. Proc Natl Acad Sci U S A.

[13] Burgie ES, Vierstra RD. Phytochromes: An Atomic Perspective on Photoactivation and Signaling. The Plant cell*.* 2014. 10.1105/tpc.114.131623 PMC4311201 25480369

[14] Yasui Y, Mukougawa K, Uemoto M, Yokofuji A, Suzuri R, Nishitani A (2012). The phytochrome-interacting vascular plant one-zinc finger1 and VOZ2 redundantly regulate flowering in Arabidopsis. Plant Cell.

[15] Nakai Y, Nakahira Y, Sumida H, Takebayashi K, Nagasawa Y, Yamasaki K (2013). Vascular plant one-zinc-finger protein 1/2 transcription factors regulate abiotic and biotic stress responses in Arabidopsis. Plant J.

[16] Yendo AC, de Costa F, Gosmann G, Fett-Neto AG (2010). Production of plant bioactive triterpenoid saponins: elicitation strategies and target genes to improve yields. Mol Biotechnol.

[17] Yousefzadi M, Sharifi M, Behmanesh M, Ghasempour A, Moyano E, Palazon J (2012). The effect of light on gene expression and podophyllotoxin biosynthesis in Linum album cell culture. Plant Physiol Biochem.

[18] Chen HQ, Wei JH, Yang JS, Zhang Z, Yang Y, Gao ZH (2012). Chemical constituents of agarwood originating from the endemic genus Aquilaria plants. Chem Biodivers.

[19] Liu YY, Chen HQ, Yang Y, Zhang Z, Wei JH, Meng H (2013). Whole-tree Agarwood-Inducing Technique: An Efficient Novel Technique for Producing High-Quality Agarwood in Cultivated Aquilaria sinensis Trees. Molecules.

[20] Ueda J, Imamura L, Tezuka Y, Tran QL, Tsuda M, Kadota S (2006). New sesquiterpene from Vietnamese agarwood and its induction effect on brain-derived neurotrophic factor mRNA expression in vitro. Bioorgan Med Chem.

[21] Chen JC, Chiu MH, Nie RL, Cordell GA, Qiu SX (2005). Cucurbitacins and cucurbitane glycosides: structures and biological activities. Nat Prod Rep.

[22] Schultz MD, Schmitz RJ, Ecker JR (2012). 'Leveling' the playing field for analyses of single-base resolution DNA methylomes. Trends Genet.

[23] Cao XF, Jacobsen SE (2002). Role of the Arabidopsis DRM methyltransferases in de novo DNA methylation and gene silencing. Curr Biol.

[24] Matzke M, Kanno T, Claxinger L, Huettel B, Matzke AJM (2009). RNA-mediated chromatin-based silencing in plants. Curr Opin Cell Biol.

[25] Henderson IR, Jacobsen SE (2007). Epigenetic inheritance in plants. Nature.

[26] Law JA, Jacobsen SE (2010). Establishing, maintaining and modifying DNA methylation patterns in plants and animals. Nat Rev Genet.

[27] Ngernprasirtsiri J, Kobayashi H, Akazawa T (1988). DNA Methylation Occurred around Lowly Expressed Genes of Plastid DNA during Tomato Fruit Development. Plant Physiol.

[28] Chellappan P, Xia J, Zhou X, Gao S, Zhang X, Coutino G (2010). siRNAs from miRNA sites mediate DNA methylation of target genes. Nucleic Acids Res.

[29] Mi SJ, Cai T, Hu YG, Chen Y, Hodges E, Ni FR (2008). Sorting of small RNAs into Arabidopsis argonaute complexes is directed by the 5 ' terminal nucleotide. Cell.

[30] Montgomery TA, Howell MD, Cuperus JT, Li DW, Hansen JE, Alexander AL (2008). Specificity of ARGONAUTE7-miR390 interaction and dual functionality in TAS3 trans-acting siRNA formation. Cell.

[31] Carthew RW, Sontheimer EJ (2009). Origins and Mechanisms of miRNAs and siRNAs. Cell.

[32] Vaucheret H (2006). Post-transcriptional small RNA pathways in plants: mechanisms and regulations. Genes Dev.

[33] Lee Y, Kim M, Han J, Yeom KH, Lee S, Baek SH (2004). MicroRNA genes are transcribed by RNA polymerase II. EMBO J.

[34] Kurihara Y, Watanabe Y (2004). Arabidopsis micro-RNA biogenesis through Dicer-like 1 protein functions. Proc Natl Acad Sci U S A.

[35] Gonzalez-Ballester D, Casero D, Cokus S, Pellegrini M, Merchant SS, Grossman AR (2010). RNA-seq analysis of sulfur-deprived Chlamydomonas cells reveals aspects of acclimation critical for cell survival. Plant Cell.

[36] Dunoyer P, Brosnan CA, Schott G, Wang Y, Jay F, Alioua A (2010). An endogenous, systemic RNAi pathway in plants. Embo J.

[37] Zhang XY, Henderson IR, Lu C, Green PJ, Jacobsen SE (2007). Role of RNA polymerase IV in plant small RNA metabolism. Proc Natl Acad Sci U S A.

[38] Ghildiyal M, Zamore PD (2009). Small silencing RNAs: an expanding universe. Nat Rev Genet.

[39] Xie ZX, Johansen LK, Gustafson AM, Kasschau KD, Lellis AD, Zilberman D (2004). Genetic and functional diversification of small RNA pathways in plants. Plos Biol.

[40] Wu L, Zhou HY, Zhang QQ, Zhang JG, Ni FR, Liu C (2010). DNA Methylation Mediated by a MicroRNA Pathway. Mol Cell.

[41] Chen DJ, Meng YJ, Yuan CH, Bai L, Huang DL, Lv SL (2011). Plant siRNAs from introns mediate DNA methylation of host genes. RNA.

[42] Wang X, Duan CG, Tang K, Wang B, Zhang H, Lei M (2013). RNA-binding protein regulates plant DNA methylation by controlling mRNA processing at the intronic heterochromatin-containing gene IBM1. Proc Natl Acad Sci U S A.

[43] Diener AC, Li H, Zhou W, Whoriskey WJ, Nes WD, Fink GR (2000). Sterol methyltransferase 1 controls the level of cholesterol in plants. Plant Cell.

[44] Draper N, Stewart PM (2005). 11beta-hydroxysteroid dehydrogenase and the pre-receptor regulation of corticosteroid hormone action. J Endocrinol.

[45] Tang Q, Ma X, Mo C, Wilson IW, Song C, Zhao H (2011). An efficient approach to finding Siraitia grosvenorii triterpene biosynthetic genes by RNA-seq and digital gene expression analysis. BMC Genomics.

[46] Lifton RP, Dluhy RG, Powers M, Rich GM, Gutkin M, Fallo F (1992). Hereditary hypertension caused by chimaeric gene duplications and ectopic expression of aldosterone synthase. Nat Genet.

[47] Miura K, Yasuda K, Yanase T, Yamakita N, Sasano H, Nawata H (1996). Mutation of cytochrome P-45017 alpha gene (CYP17) in a Japanese patient previously reported as having glucocorticoid-responsive hyperaldosteronism: With a review of Japanese patients with mutations of CYP17. J Clin Endocr Metab.

[48] He ML, Qi SY, Hu LJ (2005). Rapid in vitro propagation of medicinally important Aquilaria agallocha. J Zhejiang Univ Sci B.

[49] Li H, Durbin R (2009). Fast and accurate short read alignment with Burrows-Wheeler transform. Bioinformatics.

[50] Roberts A, Pachter L (2013). Streaming fragment assignment for real-time analysis of sequencing experiments. Nat Methods.

[51] Robinson MD, McCarthy DJ, Smyth GK (2010). edgeR: a Bioconductor package for differential expression analysis of digital gene expression data. Bioinformatics.

[52] Maere S, Heymans K, Kuiper M (2005). BiNGO: a Cytoscape plugin to assess overrepresentation of gene ontology categories in biological networks. Bioinformatics.

[53] Sun D, Xi Y, Rodriguez B, Park HJ, Tong P, Meong M (2014). MOABS: model based analysis of bisulfite sequencing data. Genome Biol.

[54] Xu Y, Zhang Z, Wang M, Wei J, Chen H, Gao Z (2013). Identification of genes related to agarwood formation: transcriptome analysis of healthy and wounded tissues of Aquilaria sinensis. BMC Genomics.

