# Supplementary material for: The effect of red light and far-red light conditions on secondary metabolism in Agarwood
Source: BMC Plant Biol. 2015 Jun 12;15:139. doi: 10.1186/s12870-015-0537-y (PMC4464252; doi:10.1186/s12870-015-0537-y)
Supplement: Additional file 2: Table S2. — (a) Whole-genome bisulfite sequencing DNA libraries and (b) sRNA sequencing libraries. Table S3. Spectral data of lamps used for different light conditions in this study. Table S4. Gene specific primers for real-time PCR analysis of gene expression. Figure S1. Gene Ontology classifications of the set of transcripts in cluster 3 and cluster 11. Relative gene proportions were calculated separately for Biological Process and Molecular Function. Figure S2. The composition of sRNAs that mapped to the A. agallocha genome. Only sRNAs which mapped perfectly and uniquely to one genome location were retained for analysis. Figure S3. Gene Ontology classifications of hyper and hypo differentially methylated regions. Relative gene proportions were calculated separately for Biological Process and Molecular Function. The set of metabolic process genes containing hypo-methylated regions were curated for secondary metabolic function and sRNA which mapped to hypo-DMR regions. Figure S4. qRT-PCR validation of mRNA expression and sRNA expression. Expression quantification from sequencing data as FPKM and TPM of the mRNA and sRNA expression are also shown, respectively. [file 12870_2015_537_MOESM2_ESM.pptx]

## Slide 1
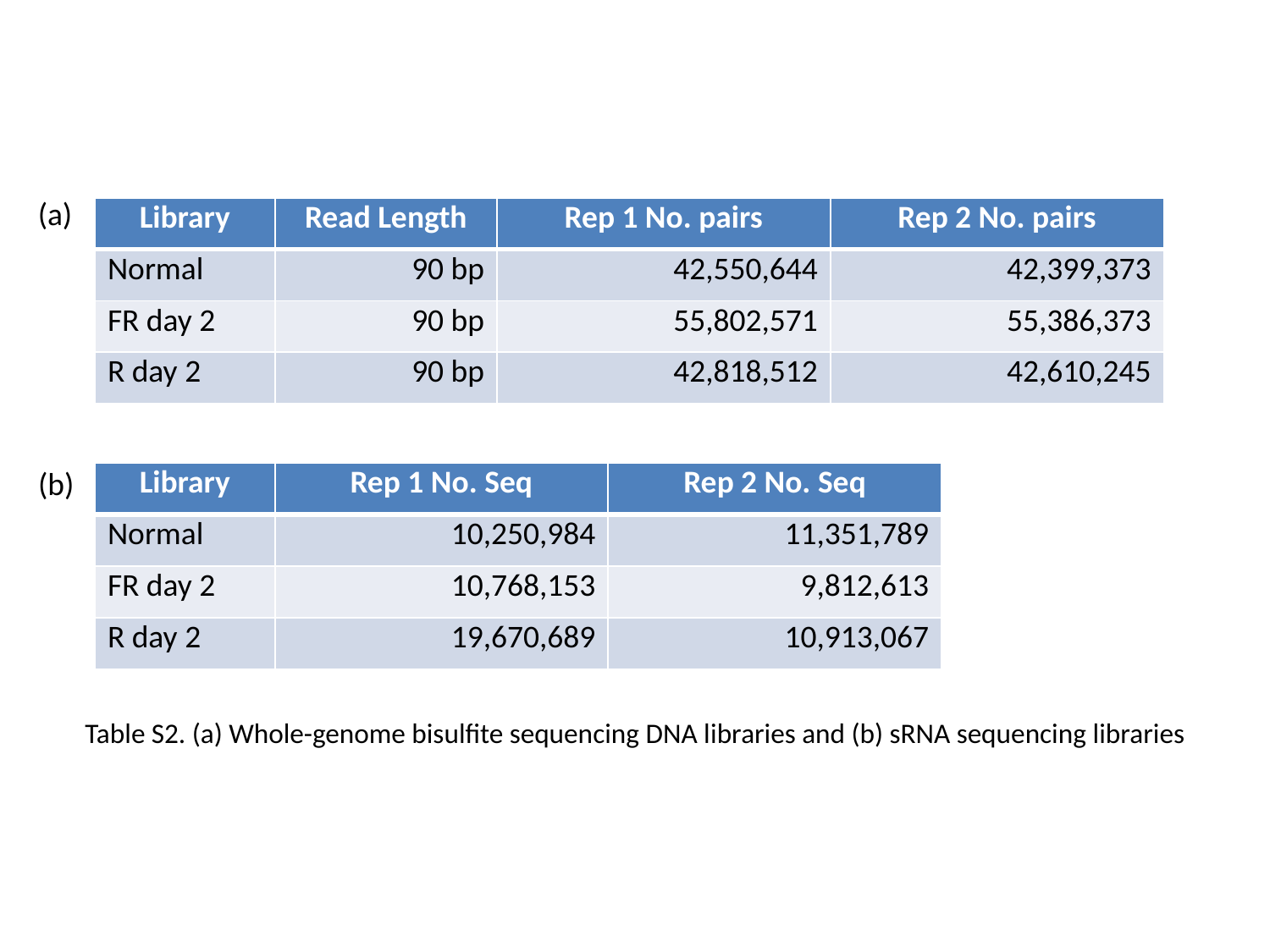

(a)
| Library | Read Length | Rep 1 No. pairs | Rep 2 No. pairs |
| --- | --- | --- | --- |
| Normal | 90 bp | 42,550,644 | 42,399,373 |
| FR day 2 | 90 bp | 55,802,571 | 55,386,373 |
| R day 2 | 90 bp | 42,818,512 | 42,610,245 |
(b)
| Library | Rep 1 No. Seq | Rep 2 No. Seq |
| --- | --- | --- |
| Normal | 10,250,984 | 11,351,789 |
| FR day 2 | 10,768,153 | 9,812,613 |
| R day 2 | 19,670,689 | 10,913,067 |
Table S2. (a) Whole-genome bisulfite sequencing DNA libraries and (b) sRNA sequencing libraries

## Slide 2
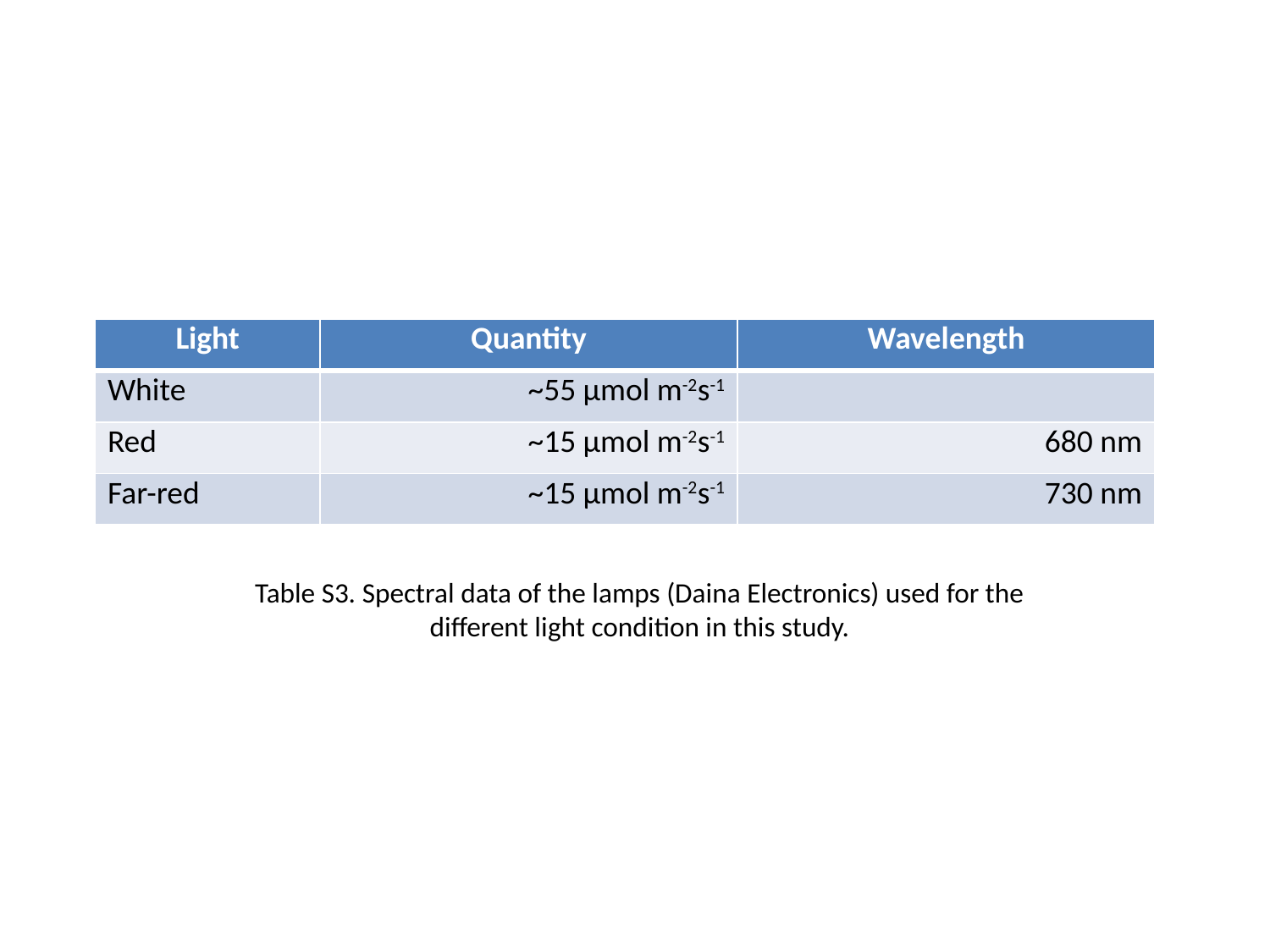

| Light | Quantity | Wavelength |
| --- | --- | --- |
| White | ~55 μmol m-2s-1 | |
| Red | ~15 μmol m-2s-1 | 680 nm |
| Far-red | ~15 μmol m-2s-1 | 730 nm |
Table S3. Spectral data of the lamps (Daina Electronics) used for the different light condition in this study.

## Slide 3
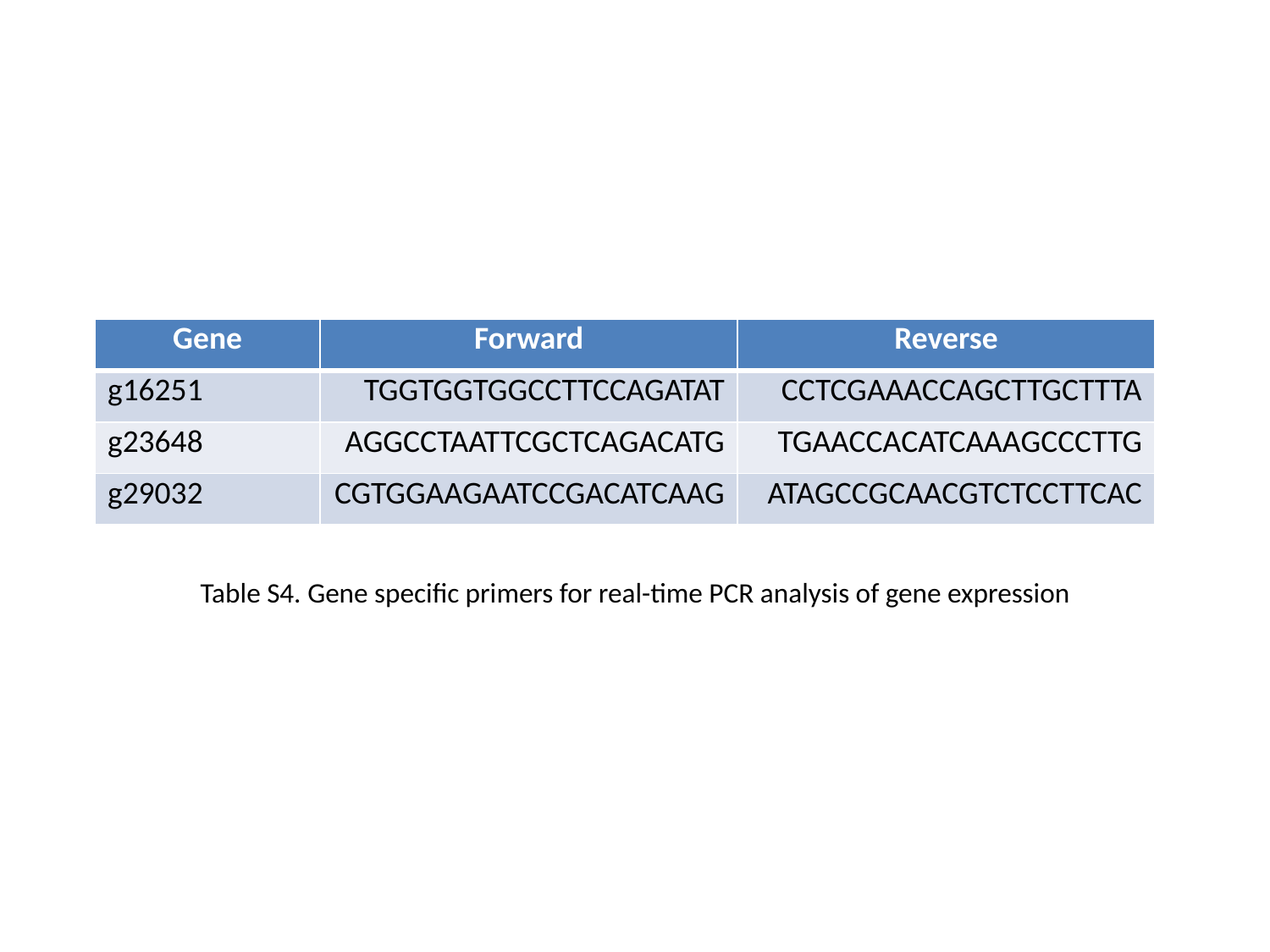

| Gene | Forward | Reverse |
| --- | --- | --- |
| g16251 | TGGTGGTGGCCTTCCAGATAT | CCTCGAAACCAGCTTGCTTTA |
| g23648 | AGGCCTAATTCGCTCAGACATG | TGAACCACATCAAAGCCCTTG |
| g29032 | CGTGGAAGAATCCGACATCAAG | ATAGCCGCAACGTCTCCTTCAC |
Table S4. Gene specific primers for real-time PCR analysis of gene expression

## Slide 4
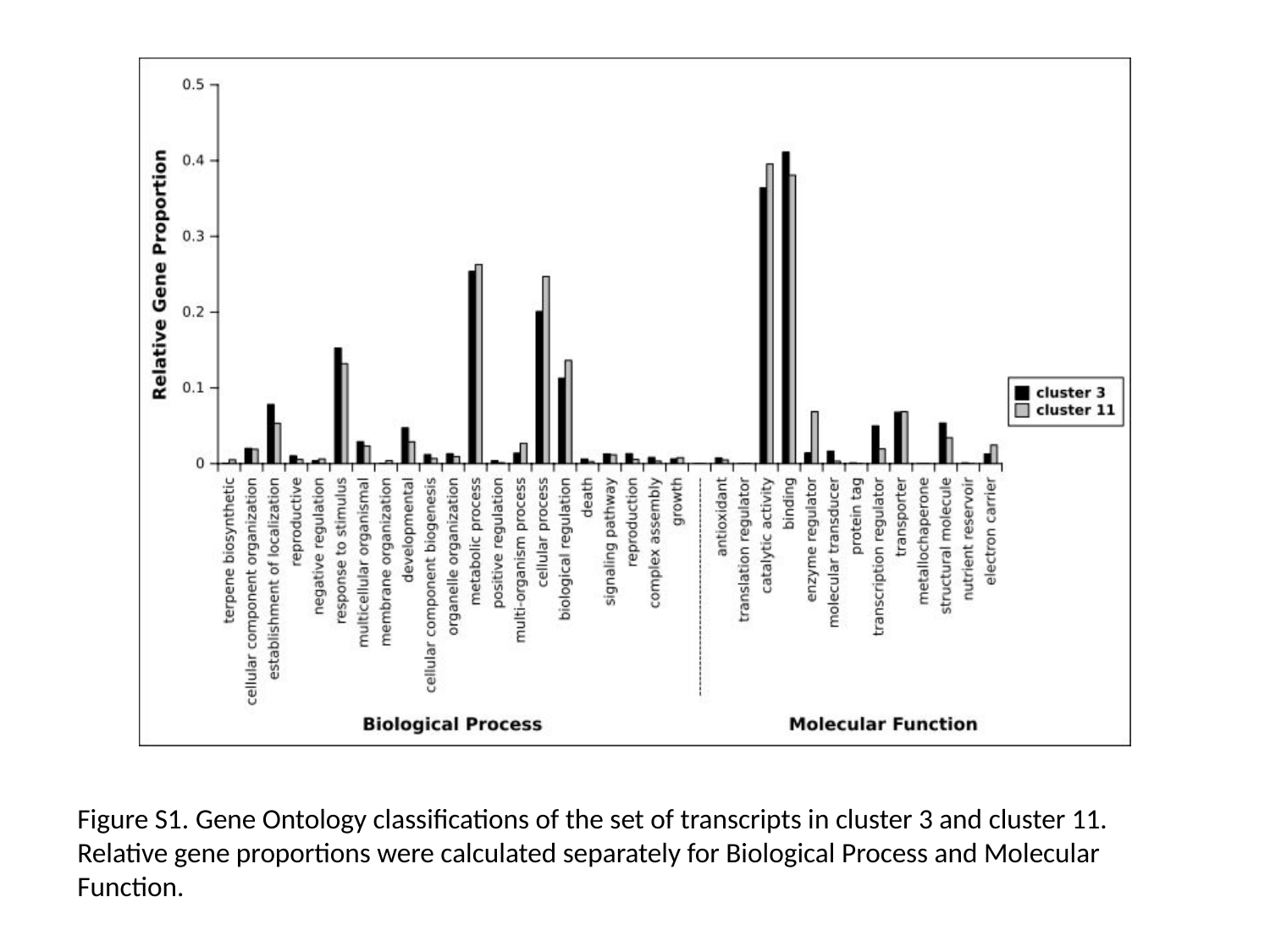

Figure S1. Gene Ontology classifications of the set of transcripts in cluster 3 and cluster 11. Relative gene proportions were calculated separately for Biological Process and Molecular Function.

## Slide 5
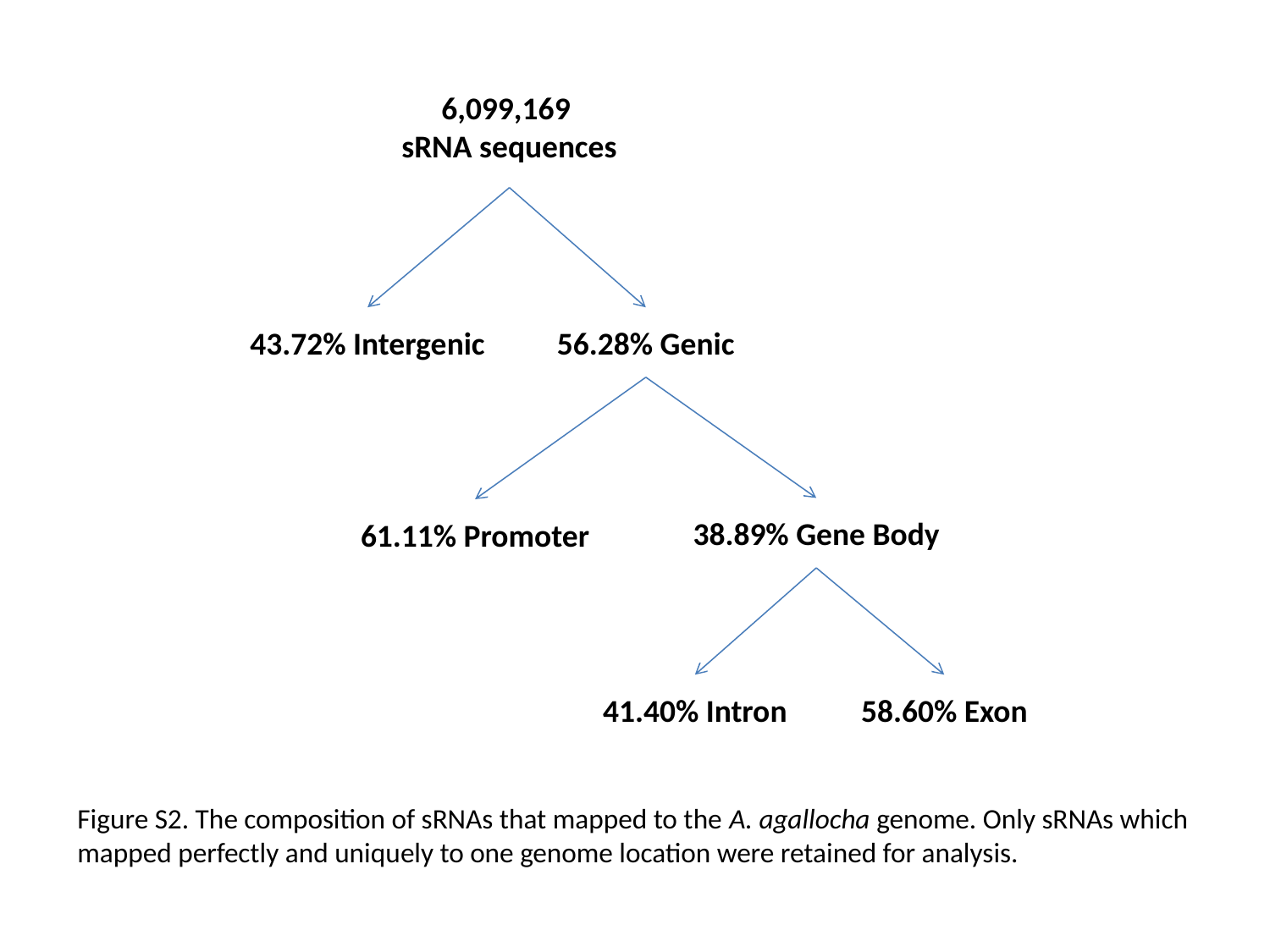

6,099,169
sRNA sequences
43.72% Intergenic
56.28% Genic
38.89% Gene Body
61.11% Promoter
41.40% Intron
58.60% Exon
Figure S2. The composition of sRNAs that mapped to the A. agallocha genome. Only sRNAs which mapped perfectly and uniquely to one genome location were retained for analysis.

## Slide 6
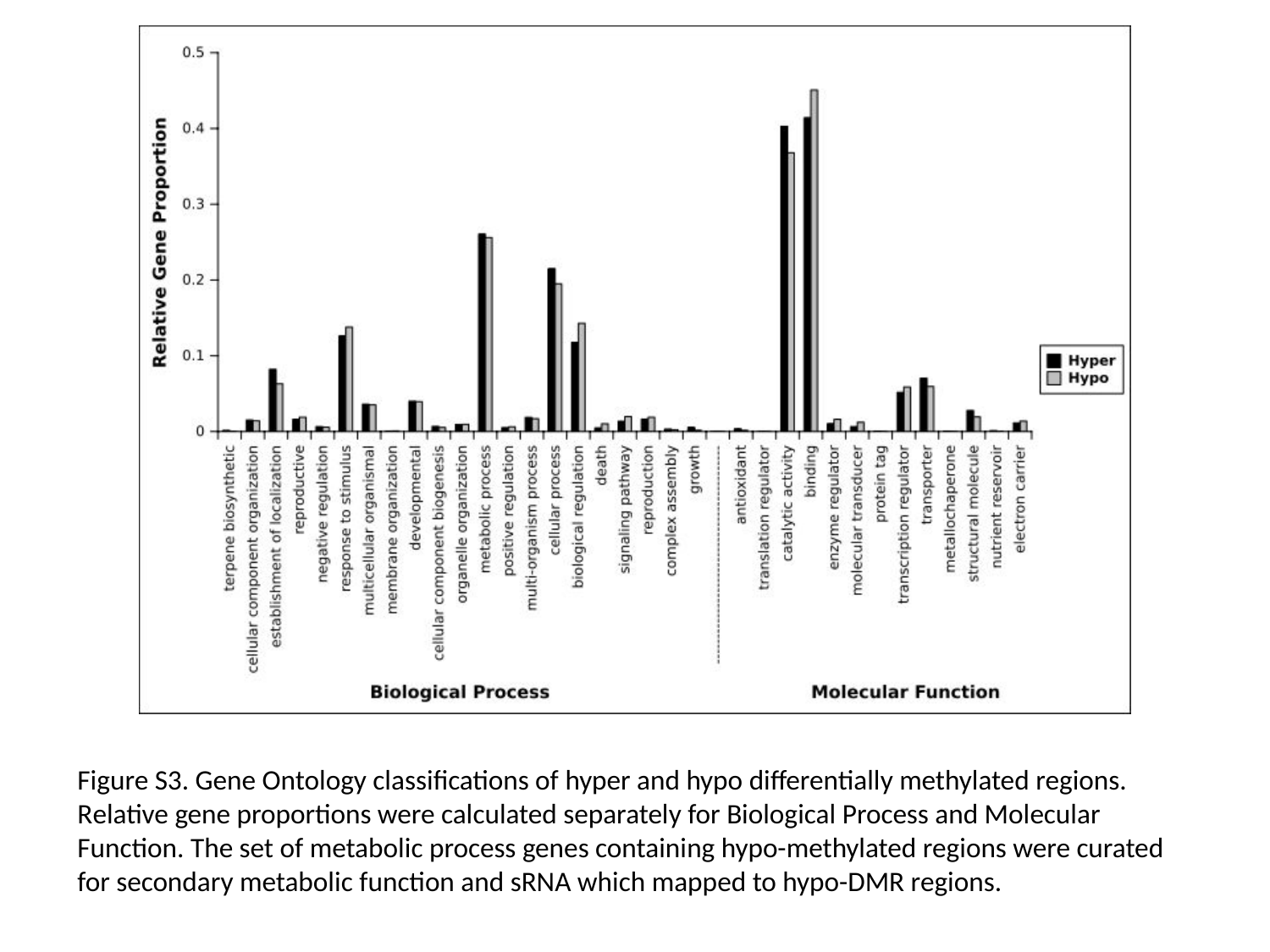

Figure S3. Gene Ontology classifications of hyper and hypo differentially methylated regions. Relative gene proportions were calculated separately for Biological Process and Molecular Function. The set of metabolic process genes containing hypo-methylated regions were curated for secondary metabolic function and sRNA which mapped to hypo-DMR regions.

## Slide 7
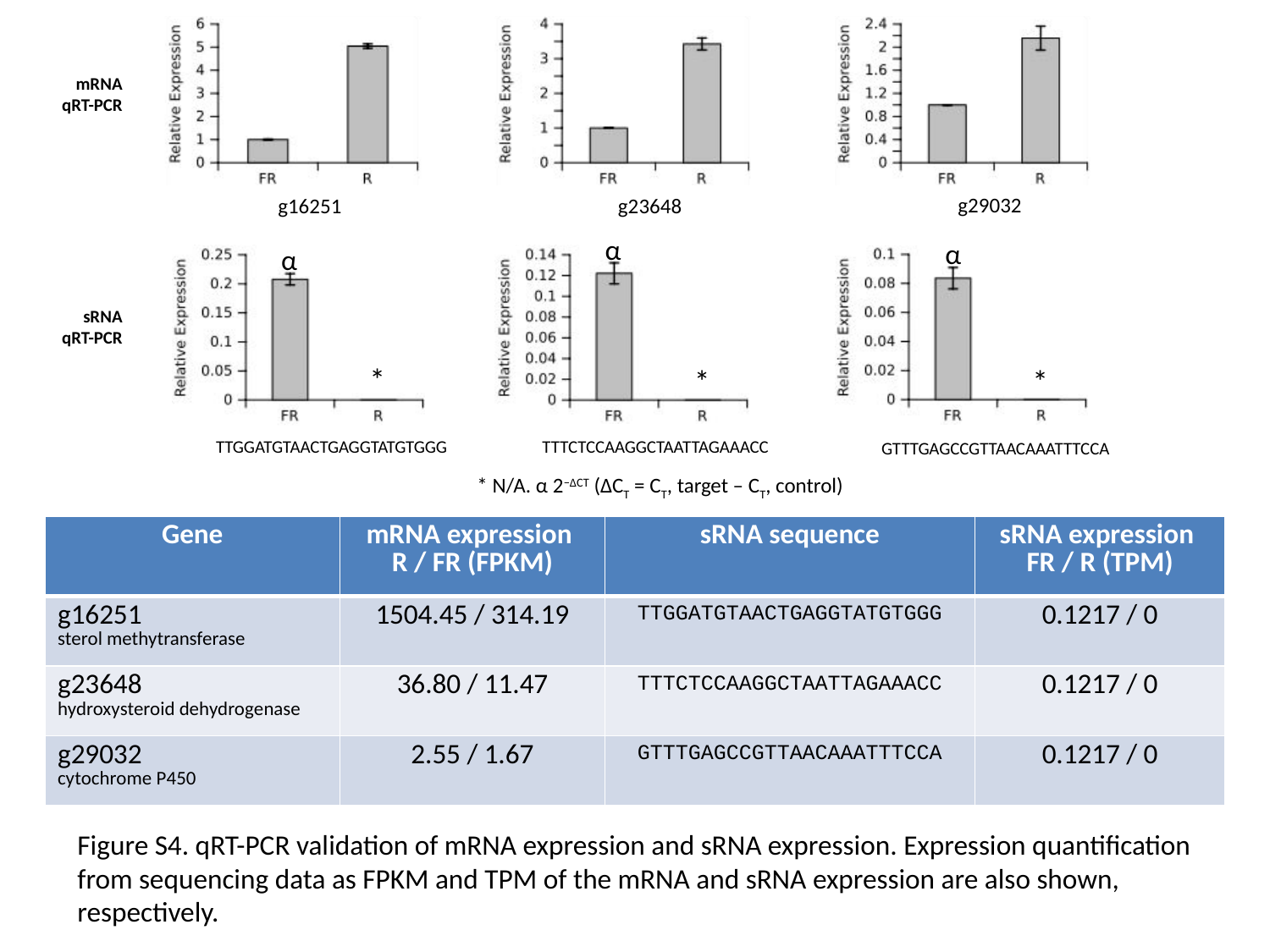

mRNA
qRT-PCR
g29032
g16251
g23648
α
α
α
sRNA
qRT-PCR
*
*
*
TTGGATGTAACTGAGGTATGTGGG
TTTCTCCAAGGCTAATTAGAAACC
GTTTGAGCCGTTAACAAATTTCCA
* N/A. α 2−ΔCT (ΔCT = CT, target – CT, control)
| Gene | mRNA expression R / FR (FPKM) | sRNA sequence | sRNA expression FR / R (TPM) |
| --- | --- | --- | --- |
| g16251 sterol methytransferase | 1504.45 / 314.19 | TTGGATGTAACTGAGGTATGTGGG | 0.1217 / 0 |
| g23648 hydroxysteroid dehydrogenase | 36.80 / 11.47 | TTTCTCCAAGGCTAATTAGAAACC | 0.1217 / 0 |
| g29032 cytochrome P450 | 2.55 / 1.67 | GTTTGAGCCGTTAACAAATTTCCA | 0.1217 / 0 |
Figure S4. qRT-PCR validation of mRNA expression and sRNA expression. Expression quantification from sequencing data as FPKM and TPM of the mRNA and sRNA expression are also shown, respectively.
